# The analysis of cannabinoids in cannabis samples by supercritical fluid chromatography and ultra‐high‐performance liquid chromatography: A comparison study

**DOI:** 10.1002/ansa.202000091

**Published:** 2020-11-26

**Authors:** Riccardo Deidda, Cédric Schelling, Jean‐Marc Roussel, Amandine Dispas, Charlotte De Bleye, Éric Ziemons, Philippe Hubert, Jean‐Luc Veuthey

**Affiliations:** ^1^ Laboratory of Pharmaceutical Analytical Chemistry University of Liège (ULiège) CIRM Liège Belgium; ^2^ School of Pharmaceutical Sciences University of Geneva Geneva Switzerland; ^3^ Institute of Pharmaceutical Sciences of Western Switzerland University of Geneva Geneva Switzerland; ^4^ Consultant Mâcon France; ^5^ Laboratory of Medicine Analysis CIRM University of Liège Liège Belgium

**Keywords:** cannabinoids, cannabis, robustness, SFC, UHPLC

## Abstract

The aim of this work was to develop a supercritical fluid chromatographic method to study the applicability of this emerging technique to cannabinoid analysis and showcase its advantages. During method development, the authors focused on nine phyto‐cannabinoids to assess the selectivity needed to potentially perform the quantitation of each cannabinoid. After method development, robustness studies were carried out on this method to gain more information about its qualitative behavior (in terms of critical resolutions) when varying some crucial parameters (concentration of additive, column temperature, starting gradient conditions and column batch). Once the robustness was evaluated and the parameters most affecting the selected responses were individuated, the SFC method was applied for a simulated routine use to generate quantitative results concerning the concentrations of the main cannabinoids in real cannabis samples. The samples were also analyzed by means of an ultra‐high‐performance liquid chromatographic method currently used in the laboratory for the same objective. Finally, the results obtained with both analytical methods were compared to evaluate their accordance. The Bland–Altman method was applied as a statistical strategy to evaluate the degree of accordance between the results generated and display the data in a difference plot. The ultra‐high performance supercritical fluid chromatography quantitative results were in accordance with the ultra‐high performance liquid chromatography results, demonstrating the applicability of this technique for cannabinoid analysis.

**Abbreviations**
1‐AA: 1‐aminoanthracene.
ABPR: active back pressure regulator
AQbD: analytical quality by design
CBC: cannabichromene
CBD: cannabidiol
CBDA: cannabidiolic acid
CBG: cannabigerol
CBGA: cannabigerolic acid
CBN: cannabinol
CMAs: critical method attributes
CMPs: critical method parameters
CNBs: cannabinoids
Col: column batch
EP: ethyl‐pyridine
FTN: flow through needle
Grad: starting gradient conditions
HCOOH: concentration of formic acid
LLOD: lower limit of detection
LLOQ: lower limit of quantification
LoA: limit of agreement
QbT: quality by testing
SFC: supercritical fluid chromatography
Temp: column temperature
THC: ∆^9^‐tetrahydrocannabinol
THCA‐A: tetrahydrocannabinolic acid
UHPLC: ultra‐high performance liquid chromatography
UHPSFC: ultra‐high performance supercritical fluid chromatography

## 1 INTRODUCTION

Cannabinoids (CNBs) present in plant material such as *Cannabis sativa* L. have been the subject of several studies in medicine, as well as in toxicology, for many years since these compounds possess different therapeutic and psychoactive properties.^1^, ^2^, ^3^, ^4^ It is worth noting that cannabis has been cultivated worldwide for centuries for its textile, medicinal and recreational use, and one of its components, the well‐known ∆^9^‐tetrahydrocannabinol (THC), remains the most widely trafficked drug in the forms of herbs, resins or oils.^1^

The main substances of interest in cannabis plants are the phyto‐cannabinoids, among them THC, but other compounds are present in the plant material, such as terpenes, flavonoids and phenolic derivatives, which possess health‐potent properties by themselves or can act synergistically with other CNBs.^4^ Today, more than 90 CNBs have been determined and can be classified into 10 subclasses.^5^, ^6^ The most important substances belonging to CNBs are THC, cannabidiol (CBD), cannabinol (CBN), and to a lesser extent, cannabigerol (CBG), and cannabichromene (CBC). These compounds, and more particularly THC and CBD used alone or in combination, present potential pharmacological properties for the treatment of different diseases, such as multiple sclerosis, cancer and chronic pain, as well as epilepsy and anxiety disorder.^2^, ^4^, ^7^ Furthermore, CBD possesses some pharmacological properties but lacks psychotropic properties, unlike THC.^7^, ^8^ Therefore, some countries have legalized cannabis for therapeutic use, and in 2018, the FDA approved Epidiolex^®^ as a drug containing cannabidiol, while Sativex^®^, a combination of THC and CBD, is currently prescribed in several countries (but not in the USA). However, the legislation is quite complex and varies by country depending on the level of THC present in the drug product. Moreover, some countries such as Uruguay and Canada have also legalized cannabis for recreational use.^8^, ^9^

Therefore, there is great interest to analyze CNBs in different matrices, such as plant extracts, herbs, oils, resins, drug formulations, drug seizures, and biological matrices. In 2018, Citti *et al* published a review on this topic, particularly on the pharmaceutical and biomedical analysis of CNBs.^3^ As mentioned in the literature, CNBs are not present in the plant in their neutral active forms because the biosynthetic route produces carboxylated species such as cannabidiolic acid (CBDA) and tetrahydrocannabinolic acid (THCA‐A), which are the most abundant acid CNBs in cannabis inflorescence.^1^, ^3^, ^5^, ^6^ The acidic precursors of all CNBs can be decarboxylated via exposure to light or heat into their neutral bioactive components. Thus, it is often necessary to determine both neutral and acidic forms in plant materials and cannabis‐derived products to obtain the total amount of a single cannabinoid, as already reported in the literature.^3^, ^9^, ^10^, ^11^, ^12^

Different chromatographic‐based methods can be used to analyze CNBs and terpenes.^1^, ^3^, ^12^, ^13^, ^14^, ^15^ For the latter, gas chromatography combined with a flame ionization detector or mass spectrometry is considered to be the gold‐standard technique, while liquid chromatography coupled with UV or MS detection is mainly used for CNBs because decarboxylation of acidic forms does not occur at ambient temperature, and no derivatization is required.^16^ The use of UV detection is surely the most widespread because all CNBs possess chromophores in their structures, and the required sensitivity is in accordance with this detection mode.^3^ Furthermore, the use of ultra‐high‐performance liquid chromatography (UHPLC) with sub‐2‐µm particles or superficially porous particles has dramatically increased the chromatographic performance in terms of analysis time, peak capacity and sensitivity.^9^, ^11^, ^14^, ^17^

In addition to the consolidated UHPLC, ultrahigh performance supercritical fluid chromatography (UHPSFC) has recently gained attention from the scientific community in the field of pharmaceutical analysis. Indeed, this latter method, presenting many advantages, such as versatility, short analysis times, and lower amounts of organic solvents needed for the mobile phases, is considered to be a valid alternative to UHPLC.^18^ In the scientific literature, only a few papers have shown the development of supercritical fluid chromatographic (SFC) methods for dosing CNBs in cannabis samples.^19^, ^20^, ^21^ To the contrary, some application notes coming from the main industrial manufactures are available. However, none of them have compared the obtained analytical performance with that of the actual gold‐standard method based on UHPLC, nor have routine application results been demonstrated.

In this context, the aim of this work was first to develop a UHPSFC‐UV method to separate and quantify the CNBs of interest in real cannabis samples. After method development, robustness studies were conducted on the UHPSFC method to gain more information regarding its behavior when varying some crucial parameters. Last, the method was applied for the analysis of 92 cannabis samples to simulate a routine application. The same samples were also analyzed by a UHPLC method to compare the quantitative results coming from both methods and evaluate the possibility of implementing UHPSFC for future routine analyses.

## 2 MATERIALS AND METHODS

### 2.1 Chemicals and reagents

Methanol, ethanol, 2‐propanol, butanol, and acetonitrile of OPTIMA LC‐MS grade, as well as water of UHPLC‐MS grade, were purchased from Fischer Scientific (Loughborough, UK). Pressurized liquid carbon dioxide, 4.5 grade (99.995%), was purchased from PanGas (Dagmerstellen, Switzerland). Formic acid was obtained from Biosolve (Valkenswaald, Netherlands).

All phyto‐cannabinoid standard solutions at 1 mg/mL in EtOH (CBG, Δ^8^‐THC, Δ^9^‐THC), in MeOH (CBC, CBN, CBD), in ACN (CBDA, CBGA), and in 2‐PrOH (THCA‐A) used for this study were obtained from Lipomed AG (Arlesheim, Switzerland).

### 2.2 Standard solutions and preparation of cannabis samples

The standard stock solution A for THC, THCA‐A, CBD, and CBDA was prepared at a concentration of 250 µg/mL (thus containing 25% of EtOH, 2‐PrOH, MeOH, and ACN). The standard stock solution B for CBN, CBG, CBGA, Δ^8^‐THC, and CBC was prepared at a concentration of 200 µg/mL (containing 40% of MeOH, 40% of EtOH, and 20% of ACN). All stock solutions were stored at ‐20°C.

Cannabis samples, provided by the School of Criminal Justice of the University of Lausanne, Switzerland, consisted of police seizures in the form of plant inflorescences and resins.

Sample preparation consisted of a solid–liquid extraction using ethanol as extraction solvent. To extract CNBs from plant material, the latter was first grossly cleared of the stem and the seeds, if present. Then, 10 mL of ethanol was added to 500 mg of plant material in an Ika ultra tube drive system for agitation and grinding for 4 min at 6000 rpm with two glass beads 6 mm in diameter. The mixture was left at ambient temperature for 9 min, and a centrifugation was carried out for 3 min at 14 000 rpm. The supernatant was diluted 50 times before injection in the chromatographic systems. For UHPLC analysis, water‐ACN (3/7, v/v) was used as the dilution solvent, while ACN was chosen for UHPSFC analyses. This extraction protocol has been previously optimized, and the robustness was tested by using a multivariate approach (data not reported).

### 2.3 UHPLC: Apparatus and methodology

#### 2.3.1 UHPLC‐UV equipment

All experiments were performed on a Waters Acquity UPLC H‐class system (Waters, Milford, MA, USA) equipped with a quaternary solvent manager, a sample manager with flow through needle (FTN) injector, and a column manager. A mix of ACN, EtOH, and water (4:4:2 v/v) was used as the wash solvent, and a mixture of ACN and water (7:3 v/v) was used as the purge solvent, with a post inject wash of 6 s. The chromatographic system was used with a Waters PDA detector for UV detection set at 214 nm in a compensated mode (compensation reference of 350–410 nm).

#### 2.3.2 UHPLC‐UV method conditions

The separation was performed at 30°C on an InfinityLab Poroshell 120 EC‐C18 column (150 × 2.1 mm, 2.7 µm) from Agilent (Santa Clara, USA) with an InfinityLab Poroshell 120 EC‐C18 guard column (5 × 2.1 mm, 2.7 µm) from Agilent. The mobile phase A contained water with 0.1% formic acid, and the mobile phase B was ACN with 0.1% formic acid. The gradient profile was the following: 2.8 min in isocratic mode with 68% of B, increased from 68% to 73% in 0.5 min, held for 3.7 min, then increased to 95% in 5.0 min, and held for 1.0 min. The percentage of B was finally brought to the initial conditions in 0.5 min and maintained for 4.5 min to re‐equilibrate the system. The flow rate was set at 0.5 mL min^−1^, and the injection volume was 1 µL.

#### 2.3.3 Calibration solutions

Standard stock solution A (containing CBD, THC, THCA‐A and CBDA) was diluted in water‐ACN (3/7, v/v) to obtain the final concentrations of 7.81, 15.62, 31.25, 62.50, and 125.00 µg/mL for each analyte.

Standard stock solution B (containing CBN, CBG, CBGA, Δ^8^‐THC, and CBC) was diluted in water‐ACN (3/7, v/v) to obtain the final concentrations of 3.12, 6.25, 12.50, 25.00, and 50.00 µg/mL for each analyte.

### 2.4 UHPSFC‐UV: Apparatus and methodology

#### 2.4.1 UHPSFC‐UV equipment

All experiments were performed on a Waters Acquity UPC^2^ system (Waters) equipped with a binary solvent manager delivery pump; a sample manager autosampler, which included a 10‐µL loop for partial loop injection; a column oven; and a two‐step (active and passive) backpressure regulator (ABPR). 2‐Propanol and methanol/water 50:50, v/v, were used as the weak and strong solvents, respectively, with volumes of 600 and 200 µL, respectively. The chromatographic system was combined with a Waters PDA detector set at 214 nm for UV detection in a compensated mode (compensation reference of 350–410 nm).

#### 2.4.2 Column screening

Three different SFC‐dedicated analytical columns provided by Waters were tested during the method development: Acquity UPC^2^ Torus Diol, Torus 1‐AA (1‐aminoanthracene) and Viridis BEH‐2EP (ethyl‐pyridine). All selected columns were of the same dimensions (100 × 3.0 mm) and particle size (1.7 µm).

The mobile phase was composed of carbon dioxide and a mix of MeOH/water (98/2 v/v) as the modifier, and the flow rate was set at 1.5 mL/min. The temperature of the column was maintained at 40°C, and the backpressure was kept constant at 120 bar. A generic gradient was employed starting with 2% of modifier up to 45% in 3.5 min, followed by an isocratic step of 1 min. The column was reconditioned to initial conditions in 0.5 min and was maintained for 2 min to re‐equilibrate the column before the subsequent injections.

#### 2.4.3 Final conditions

The final chromatographic conditions were applied to cannabis extracts using an Acquity UPC^2^ Torus Diol column (100 × 3.0 mm, 1.7 µm). The selected organic modifier was 2‐propanol with 0.1% of formic acid, and the following gradient was used: the initial conditions were 2.5% of organic modifier with a subsequent linear increase to 12% in 3 min, which was then increased to 25% in 1 min, and the organic modifier was then brought back to 2.5% in 1 min. The system was finally re‐equilibrated for 1 min prior to the subsequent injections. The flow rate was set at 1.5 mL/min, the injection volume was 1 µL, the column temperature was kept at 45°C, and the backpressure was kept constant at 120 bar.

#### 2.4.4 Calibration solutions

Standard stock solution A (containing CBD, THC, THCA‐A and CBDA) was diluted in ACN to obtain the final concentrations of 7.81, 15.62, 31.25, 62.50, and 125.00 µg/mL for each analyte.

Standard stock solution B (containing CBN, CBG, CBGA, Δ^8^‐THC and CBC) was diluted in ACN to obtain the final concentrations of 3.12, 6.25, 12.50, 25.00, and 50.00 µg/mL for each analyte.

### 2.5 Robustness studies

Robustness studies for the UHPSFC method were conducted on a mix standard solution containing the nine CNBs of interest: CBD, Δ^8^‐THC, THC, CBC, CBN, THCA‐A, CBDA, CBG, and CBGA. The concentration for CBDA, CBD, THCA‐A, and THC was 50 µg/mL, while it was 9 µg/mL for the other compounds. These concentrations were chosen to simulate those found in real cannabis samples.

A Full Factorial Design was chosen to generate the experimental plan. Four factors (X_1_ to X_4_), and first order interactions were considered in the model:

$$\begin{array}{c} Y\;=b_{0}\;+b_{1}X_{1}+b_{2}X_{2}+b_{3}X_{3}+b_{4}X_{4}+b_{1,\;2}\left(X_{1}X_{2}\right)+b_{1,\;3}\left(X_{1}X_{3}\right)\\ \;\;\;\;\;\;\;\;\;\;+\;b_{2,\;3}\left(X_{2}X_{3}\right)+b_{1,\;4}\left(X_{1}X_{4}\right)+b_{2,\;4}\left(X_{2}X_{4}\right)+b_{3,\;4}\left(X_{3}X_{4}\right)+\varepsilon \end{array}$$

where X_1_ is the concentration of additive in the organic modifier (*HCOOH*), X_2_ the column temperature (*Temp*), X_3_ the column batch (*Col*), and X_4_ the starting gradient conditions (*Grad*).

### 2.6 Software

The two chromatographic systems were equipped with Empower™ 3 software (Waters) that was used to control the two systems and for data acquisition.

The NemrodW software package (NemrodW, Marseille, France) was used to generate the full factorial design used to study the robustness and for data treatment.

## 3 RESULTS AND DISCUSSION

### 3.1 UHPSFC method development

**FIGURE 1:**
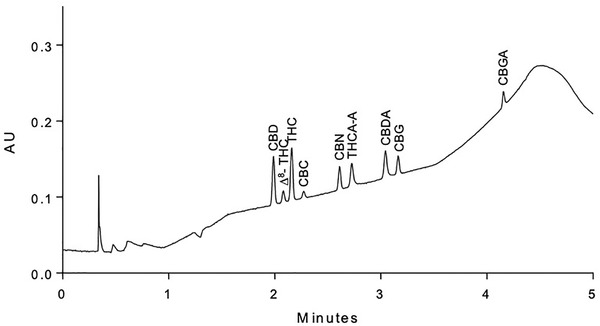
UHPSFC chromatogram referring to the analysis of the analyte mixture applying the final method conditions. Stationary phase, Acquity UPC^2^ Torus DIOL column (100 × 3.0 mm, 1.7 µm). Mobile phase, carbon dioxide (A) and 2‐propanol with 0.1% formic acid (B). Gradient conditions, the initial conditions were 2.5% of organic modifier with a subsequent linear increase to 12% in 3 min, next to 25% in 1 min and brought back to 2.5% in 1 min. The system was finally re‐equilibrated for 1 min prior the subsequent injections. Flow rate, 1.5 mL/min; injection volume, 1 µL; column temperature, 45°C; BPR, 120 bar. The concentration for CBDA, CBD, THCA‐A, and THC was 50 µg/mL while for the other compounds 9 µg/mL

Method development was performed by using the quality by testing (QbT) approach.^22^ Three different SFC‐dedicated columns were selected as the most promising stationary phases according to the chemical properties of the nine analytes. Methanol with 2% v/v of water was chosen as the modifier of carbon dioxide for the column screening. A generic linear gradient, going from 2% of organic modifier up to 45%, was applied for each column, as indicated in Section 2.4.2. Although a complete separation was not achieved, the Torus DIOL column exhibited the largest number of peaks in the chromatogram and was selected to continue with the method development. Ethanol, 2‐propanol, and *n*‐butanol were then tested as progressively longer‐chain alcohols to try to separate the critical peak pairs (CBN–THCA‐A and CBDA–CBG) and improve the selectivity. The gradient conditions were varied and tested simultaneously with the modifier. Finally, 2‐propanol was able to achieve the required selectivity to perform the quantitation of each analyte and then was selected as the final modifier. Furthermore, the addition of 0.1% formic acid to the modifier improved the peak shapes for the acidic CNB forms and then was retained as an additive. Figure 1 shows a chromatogram obtained by the analysis of a mix standard solution with the nine analytes applying the final chromatographic conditions. The separation of the nine analytes was obtained in a total runtime of 6 min. The short analysis time and the low amount of organic modifier needed to elute the last analyte make this method particularly convenient for possible routine use.

Since the analytical method was developed by using the QbT approach and the tested experimental domain was not so extensive, not much information about the qualitative method performance was collected at this stage. As a matter of fact, before applying an analytical method to generate qualitative or quantitative data, knowledge about the influence of some crucial parameters should be acquired to better control the method when applied to routine analyses. In this context, two strategies are currently used by analytical chemists, and both rely on multivariate approaches. The first is analytical quality by design (AQbD), which is focused on the notion of risk applied to the entire method life cycle, from the very beginning of method development to the control strategy, when the method performs in routine.^22^ The second approach, used in the present study, consists of performing a robustness study by means of an experimental design. Robustness is generally evaluated after method development and allows for exploration of the outline of the final conditions of the method, before a formal validation.

### 3.2 Robustness studies

Robustness of an analytical method is defined as “its capacity to remain unaffected by small but deliberate variations in method parameters” and “provides an indication of its reliability during normal usage.”^23^ This type of study also allows for the acquisition of information regarding the behavior of selected critical method attributes (CMAs) when some method parameters vary and eventually individuation of the most effective ones, also named critical method parameters (CMPs).^16^ If CMPs are individuated, appropriate precautions regarding the usage of the analytical method can be taken.

A robustness study is particularly interesting when developing an analytical method by using the QbT approach, and above all in regard to a UHPSFC method, because it has been poorly evaluated and documented in the scientific literature. Moreover, in UHPSFC, small parameter variations can have important consequences in terms of separation. Therefore, it is crucial to correctly investigate and individuate these parameters.

**FIGURE 2:**
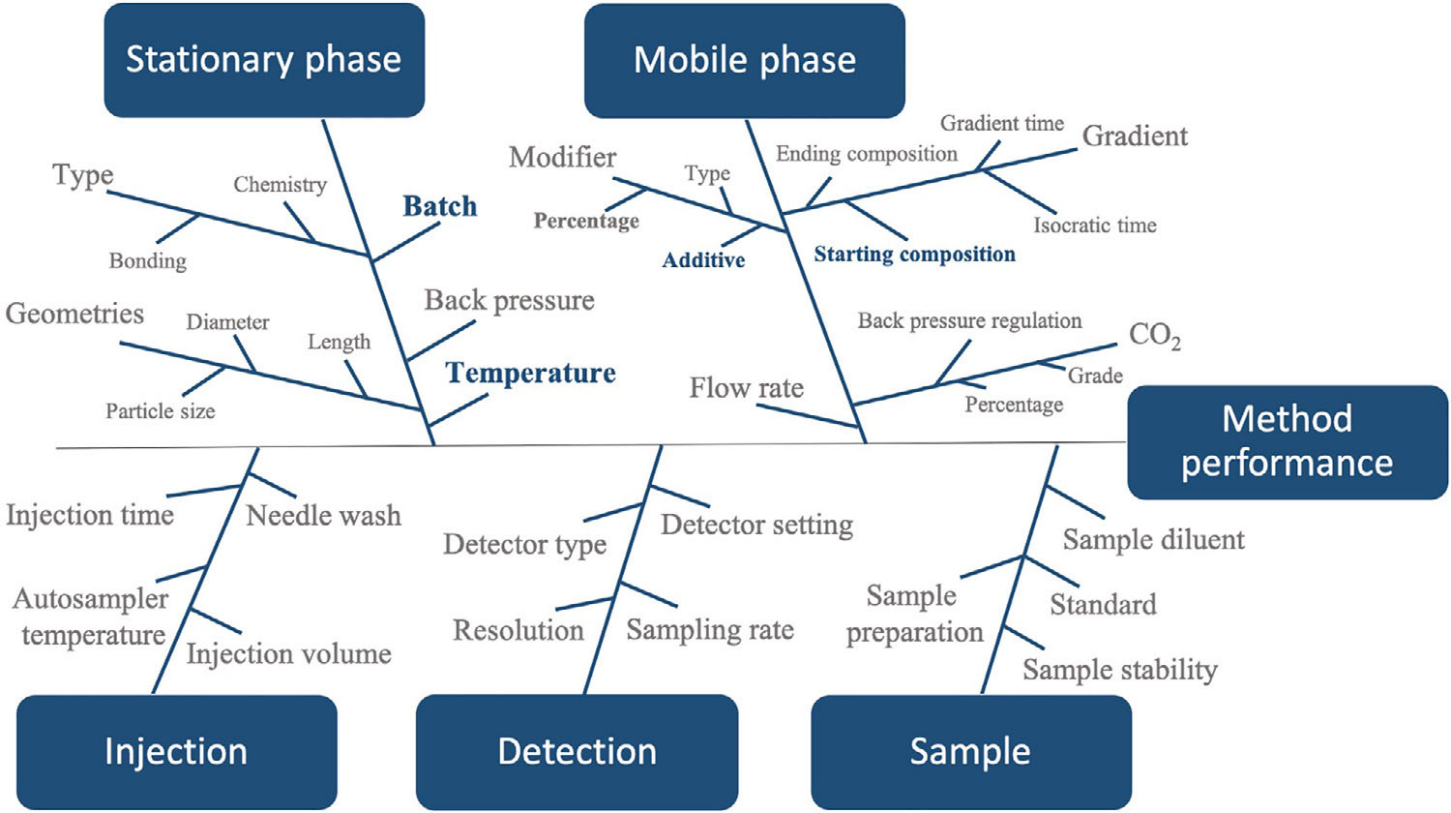
Ishikawa diagram for the UHPSFC method where method parameters are divided in 5 main groups: stationary phase, mobile phase, injection, detection, and sample. The highlighted parameters, colored in dark blue, are those selected for the study of method robustness

When evaluating robustness, the generally studied parameters are those linked to sources of experimental noise, such as instrument, analyst, and materials used to perform the analyses.^24^ In this context, an Ishikawa diagram, Figure 2, has been drawn and used as a risk assessment tool to well visualize all of the parameters potentially affecting method performance and choose those to be further investigated. Their choice was made to simulate an eventual equipment malfunction, which could occur during routine analyses, or a lack of precision related to the analyst during the preparation of the mobile phases, etc. In this context, the column temperature (*Temp*), column batch (*Col*), concentration of additive in the organic modifier (*HCOOH*), and starting gradient conditions (*Grad*) were the parameters investigated in the following range: Temperature, 40–50°C; *Col*, batches 1–2; HCOOH, 0.08–0.12%; and *Grad*, 2–3% min^−1^. Concerning the *Col* parameter, two columns coming from different batches were selected. Furthermore, to represent a possible real situation of a routine usage of the columns, the authors chose two used columns with a different number of injections already performed. Regarding the CMAs, one type of response was chosen to be studied, the USP resolution, since the possibility of accurately quantitating analytes relies on it (a *R_s_* ≥ 1.5 is needed for quantitative and qualitative purposes). All of the resolutions related to the nine analytes were calculated and monitored during the robustness study, but only 5 resolutions were classified as potentially critical: the resolutions between CBD and Δ^8^‐THC (*R_sCBD‐Δ8‐THC_*), Δ^8^‐THC and THC (*R_sΔ8‐THC‐THC_)*, THC and CBC (*R_sTHC‐CBC_)*, CBN and THCA‐A (*R_sCBN – THCA–A_*) and CBDA and CBG (*R_sCBDA – CBG_*). Therefore, only the results obtained for these responses are discussed hereafter.

A full factorial design matrix with a total of 16 runs was applied to study the effect of the selected method parameters and those of their first order interactions on the CMAs, as detailed in 2.5. The acceptable lower limit for resolution was set at *R_s_* ≥ 1.5, and all resolutions above this limit were defined as appropriate for the analytical purpose. Table 1 shows the experimental design with the values obtained for each response, including the experiments at the center of the domain; the detailed results given by the experimental design are presented in the supplementary material. For design results interpretation, two different tools have been used. First, each parameter and interaction effect has been tested for statistical significance (Student t‐test) and then compared to the desired specification (*R_s_* ≥ 1.5). It appeared from the individual effects analysis that the gradient slope has a highly significant, but acceptable, effect on all measured resolutions, while the temperature effect, also significant or highly significant on the measured resolutions, has been found over the specification limit for *R_sCBN – THCA–A_* and *R_sCBDA – CBG_*. The formic acid concentration has been found to be highly significant for *R_sCBN – THCA–A_* and *R_sCBDA – CBG_* but remains within the desired specification, and the column batch effect has been found to be significant or highly significant, but acceptable, for all measured resolutions. None of the interaction effects were found critical, the highest of them being only of 0.03 units on the corresponding resolution value. Based on these findings, method robustness was not verified for the temperature effect on *R_sCBN – THCA–A_* and *R_sCBDA – CBG_*. Some results, highlighted in red in Table 1, obtained for *R_sCBN – THCA–A_* and *R_sCBDA – CBG_* responses, show a complete loss of resolution, which cannot be explained by the temperature effect alone. Most probably, in these particular experimental conditions, a joint effect of several factors is responsible for the loss of resolution, mainly due to the temperature effect and, to a lesser extent, the formic acid content in the mobile phase and the gradient slope. Another possible explanation could lie in a correlation between the simultaneous variation in temperature and percentage of organic modifier in the mobile phase. Indeed, looking at the gradient slope and at the robustness results at the same time, one can rapidly note that, for the first eluting peak pairs, when the percentage of organic modifier is relatively low, the temperature effect on resolutions remained within the specification limit, and robustness was demonstrated for all of these responses. A lack of robustness related to the column temperature effect appeared for the first time for the CBN–THCA‐A peak pair and after for the CBDA–CBG pair, which are observed in the chromatogram when the percentage of organic modifier is about to reach 12%. Thus, the elution zone with higher percentages of modifier was shown to be the least robust, presenting a significant loss of resolution for both peak pairs, despite these two pairs having presented the highest values of resolution at the working point conditions (at the center of the experimental domain), as can be seen in the chromatogram reported in Figure 1. Changes in temperature have an impact on the selectivity, altering the interaction between the analyte and mobile/stationary phase. However, these three parameters are easily manageable with high‐quality labware and maintained and qualified equipment performing in routine. Then, particular attention has to be given to these parameters, which appeared to be critical.

**TABLE 1:** Full factorial design (Experiment no. 1–16) including the repetitions in the center of the domain (Exp. no. 17 – 22) for robustness tests conducted on the UHPSFC method

Exp. no.	*HCOOH (%)*	*Temp(°C)*	*Col(batch)*	*Grad(%)*	*R_s CBD‐Δ_^8^_‐THC_*	*R_s Δ_^8^_‐THC‐THC_*	*R_s THC ‐CBC_*	*R_s CBC‐CBN_*	*R_s CBN‐THCA‐A_*	*R_s THCA‐A‐CBDA_*	*R_s CBDA‐CBG_*	*R_s CBG‐CBGA_*
1	0.08	40	2	2	2.27	1.96	2.57	8.08	2.13	6.07	3.03	23.69
2	0.08	40	1	3	2.02	1.72	2.61	6.86	1.83	5.80	3.78	25.40
3	0.12	50	1	3	2.20	1.68	3.06	6.65	2.97	5.79	2.31	26.76
4	0.08	40	1	2	2.66	1.93	3.23	7.53	2.52	5.87	3.30	25.07
5	0.12	40	1	3	1.99	1.66	2.65	4.23	0.30	5.16	5.00	25.57
6	0.12	40	2	2	2.24	1.94	2.63	7.79	0.70	6.46	4.24	23.47
7	0.08	50	1	2	2.79	1.85	3.74	7.43	5.05	2.07	0.00	20.02
8	0.12	40	2	3	1.74	1.70	2.10	6.07	0.00	5.79	4.50	24.23
9	0.08	40	2	3	1.72	1.73	2.09	7.20	1.84	6.10	3.21	24.44
10	0.12	50	2	2	2.44	1.92	3.06	7.93	3.28	6.18	1.54	24.57
11	0.12	50	1	2	2.79	1.84	3.70	7.44	3.67	5.77	1.76	25.71
12	0.08	50	2	2	2.48	1.95	3.08	7.98	4.62	5.27	0.00	20.58
13	0.12	40	1	2	2.65	1.93	3.22	7.50	1.22	6.41	4.48	24.97
14	0.08	50	1	3	2.28	1.68	3.00	6.49	4.36	4.85	0.75	23.61
15	0.12	50	2	3	1.97	1.72	2.60	7.25	2.85	6.23	1.89	25.81
16	0.08	50	2	3	1.99	1.73	2.65	7.36	4.17	4.47	0.27	13.36
17	0.10	45	1	2.5	2.46	1.81	3.23	7.21	2.69	5.93	2.87	26.18
18	0.10	45	1	2.5	2.49	1.80	3.10	7.00	2.77	5.86	2.80	26.15
19	0.10	45	1	2.5	2.48	1.80	3.14	6.99	2.82	5.79	2.71	26.11
20	0.10	45	2	2.5	2.11	1.80	2.66	7.90	2.32	6.29	2.67	24.62
21	0.10	45	2	2.5	2.15	1.84	2.64	7.93	2.48	6.28	2.54	24.87
22	0.10	45	2	2.5	2.14	1.84	2.68	7.88	2.52	6.15	2.41	24.75

Exp. no, number of experiment*; HCOOH*, percentage of formic acid in the organic modifier; *Temp*, column temperature*; Col*, column batch; *Grad*, starting composition of organic modifier for the gradient; *R_s_*, USP resolution.

Robustness of the UHPLC method has also been studied in a preliminary method development step. A 2^5‐1^, 16‐experiment, fractional factorial design was used to test five parameters (column age, temperature, flow rate, formic acid content in water and formic acid content in methanol).

This study demonstrated that all individual effects were above the desired minimum specification (*R_s_* ≥ 1.5) for all tested peak pairs. One set of experimental conditions showed a loss of resolution for *R_sCBC‐THCA_*, which means, as already seen for the UHPSFC method, that, in some particular experimental conditions, method robustness could not be verified due to a joint effect of several factors. Regarding *R_sCBC‐THCA_*, this loss of resolution was found due to the column age and the temperature effects. For the UHPSFC method, these two parameters can be easily managed on a qualified instrument and by a strict column performance suitability test.

### 3.3 Comparison between UHPLC and UHPSFC

**FIGURE 3:**
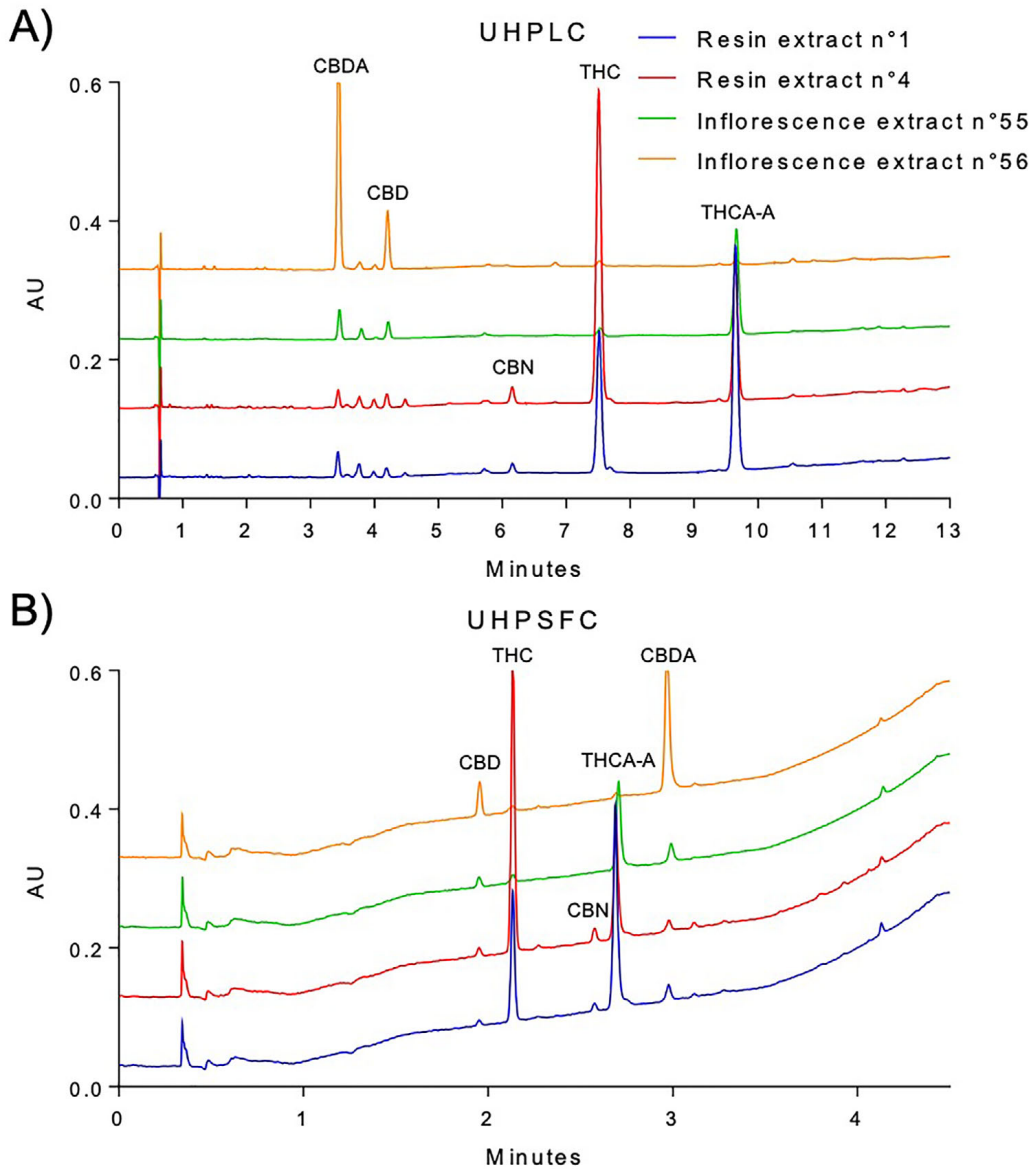
Chromatograms obtained from the analysis of two cannabis resin samples (resin extract n°1 and resin extract n°4), a “THC‐like” cannabis inflorescence (inflorescence extract n°55) and a “CBD‐like” cannabis inflorescence (inflorescence extract n°56) samples by means of the UHPLC (A, on the top) and UHPSFC (B, on the bottom) methods. Chromatographic conditions are described in Section 2.3.2 for UHPLC and in Section 2.4.3 for UHPSFC

Once the robustness was evaluated, the UHPSFC method was finally implemented for a simulated routine use. A total of 92 real cannabis samples were analyzed by both UHPLC and UHPSFC systems with the aim to quantify some of the main phyto‐cannabinoids: THC, THCA‐A, CBD, CBDA, and CBN. UV detection was chosen for these analyses since all analytes present chromophores in their structures. Moreover, the SFC‐UV hyphenation is widely spread in analytical laboratories because of its ease of use and cost‐effectiveness. The real samples consisted of both cannabis inflorescences and resins, which underwent a solid liquid extraction as detailed in Section 2.2. As an example, Figure 3 displays the chromatograms obtained from the analysis of two cannabis resin samples (resin extract n°1 and resin extract n°4) and “THC‐like” cannabis inflorescence (inflorescence extract n°55) and “CBD‐like” cannabis inflorescence (inflorescence extract n°56) samples by means of the UHPLC (A, on the top) and UHPSFC (B, on the bottom) methods. Figure 3B shows a chromatogram of a resin sample (resin extract n°1) where a problem of selectivity has been noticed for the UHPSFC method. Peak purity data from 33% of the sample set are provided in Table S4 of Supplementary. Although the selectivity was demonstrated, during method development, for the nine most common phyto‐cannabinoids found in cannabis samples, in some real samples, a minor peak next to the THCA‐A one was observed during the simulated routine use. This is a common problem when approaching a complex sample, as cannabis samples, which may potentially contain a large number of chemical compounds (just regarding CNBs, more than 90 phyto‐cannabinoids have been isolated to date and can possibly be present in real samples). It is obvious that method selectivity cannot be demonstrated in an absolute manner for this very large number of analytes with any chromatographic system in a reasonable analysis time, neither with a UHPSFC nor with a UHPLC system, but only relatively to a certain number of compounds chosen during method development or validation. In addition to this, there is also the unpredictability of this chemical profile, which can considerably vary depending on the origin of the sample. For these reasons, the use of a UHPSFC system coupled with both UV and MS detectors could help to solve this type of issue in a routine application. In fact, more selectivity could be gained thanks to the MS detector, which would allow having a specific response depending on the differences concerning mass/charge ratios of the analytes. Several studies have demonstrated that UHPSFC can be successfully coupled to MS detectors, and this chromatographic method can be easily transferred to MS detection by integrating it with an appropriate make‐up solution to promote analyte ionization (for instance, in ESI‐MS, MeOH/water 98/2% v/v with 0.1% of ammonium formate).^25^ However, some considerations must be made in this context. Indeed, although some structural differences among cannabinoids can be often observed on the alkyl chain of the phenolic ring, resulting in different mass/charge ratios, sometimes this is not the case. As an example, cannabinoids such as THC and CBD are isobaric compounds, and it is essential to separate them before the detection, as this method does.

Table 2 summarizes the quantitative results obtained for one of the considered analytes, THC, with both techniques on the totality of the samples used for this study, as well as the values obtained from the statistical data treatment performed. The results related to the other four analytes are reported in Table S2. Analyzing these results, a lack of sensitivity has been observed for the UHPSFC method in comparison to the UHPLC one when quantifying minor cannabinoids such as CBD and CBN. This point has been investigated more deeply, and the lower limits of detection (LLOD) and of quantification (LLOQ) have been estimated for both methods. LLOD is defined as the smallest analyte quantity that can be detected but not accurately quantified, while LLOQ is the smallest analyte quantity that can be accurately quantified. These parameters were estimated from the calibration line at low concentrations as follows:

$$LLOD\;or\;LLOQ\;=\frac{F*SD}{b}\;$$

where

**TABLE 2:** a) Quantitative data for THC used to evaluate the agreement between the two analytical methods, UHPLC and UHPSFC. The table includes also the true values, the relative difference percentages, the bias and the values of the limits of agreement used to obtain the Bland‐Altman plot

Cannabis sample type	Sample no	Conc UHPLC (% w/w)	Conc UHPSFC (% w/w)	True value (% w/w)	Relative difference percentage (%)
Resin	1	8.44	8.64	8.54	‐2.34
Resin	2	15.21	15.29	15.25	‐0.53
Resin	3	5.28	5.42	5.35	‐2.54
Resin	4	7.07	7.19	7.13	‐1.67
Resin	5	7.61	7.50	7.55	1.53
Resin	6	16.15	16.72	16.44	‐3.49
Resin	7	8.37	8.28	8.33	1.17
Resin	8	11.83	11.97	11.90	‐1.20
Resin	9	1.55	1.53	1.54	1.41
Resin	10	7.22	7.22	7.22	0.01
Resin	11	13.64	13.93	13.79	‐2.09
Resin	12	25.53	26.04	25.79	‐1.97
Resin	13	21.99	24.01	23.00	‐8.78
Resin	14	16.51	17.92	17.22	‐8.17
Resin	15	11.62	11.95	11.78	‐2.77
Resin	16	13.26	14.21	13.73	‐6.90
Resin	17	15.67	17.76	16.71	‐12.50
Resin	18	14.22	15.39	14.81	‐7.90
Resin	19	9.06	9.82	9.44	‐8.14
Resin	20	13.18	14.27	13.72	‐7.99
Resin	21	15.11	16.28	15.69	‐7.49
Resin	22	13.11	14.23	13.67	‐8.13
Resin	23	8.23	8.77	8.50	‐6.31
Resin	24	13.43	14.53	13.98	‐7.85
Resin	25	10.63	11.53	11.08	‐8.14
Resin	26	21.49	23.69	22.59	‐9.72
Resin	27	9.26	9.93	9.60	‐6.98
Resin	28	9.32	10.04	9.68	‐7.45
Resin	29	10.88	11.92	11.40	‐9.09
Resin	30	13.02	13.77	13.40	‐5.59
Resin	31	6.89	7.25	7.07	‐5.03
Resin	32	14.24	15.18	14.71	‐6.35
Resin	33	15.46	16.37	15.91	‐5.70
Resin	34	14.45	15.53	14.99	‐7.16
Resin	35	12.00	13.14	12.57	‐9.10
Resin	36	8.01	8.65	8.33	‐7.68
Resin	37	18.87	20.43	19.65	‐7.91
Resin	38	10.86	11.82	11.34	‐8.44
Resin	39	26.05	29.18	27.61	‐11.33
Resin	40	19.36	20.99	20.17	‐8.05
Resin	41	11.81	12.92	12.37	‐8.96
Resin	42	11.85	12.77	12.31	‐7.49
Resin	43	8.83	9.58	9.21	‐8.17
Resin	44	6.45	6.66	6.55	‐3.18
Inflorescence	45	1.07	1.07	1.07	‐0.32
Inflorescence	46	3.93	4.21	4.07	‐6.70
Inflorescence	47	2.05	2.13	2.09	‐4.00
Inflorescence	48	0.78	0.71	0.74	8.73
Inflorescence	49	–	–	–	–
Inflorescence	50	0.72	0.65	0.69	9.83
Inflorescence	51	1.59	1.60	1.60	‐1.09
Inflorescence	52	1.25	1.43	1.34	‐13.61
Inflorescence	53	1.03	0.98	1.01	4.95
Inflorescence	54	0.37	0.32	–	–
Inflorescence	55	0.47	0.53	0.50	‐11.77
Inflorescence	56	–	–	–	–
Inflorescence	57	–	–	–	–
Inflorescence	58	1.36	1.37	1.37	‐0.67
Inflorescence	59	0.57	0.54	0.55	5.98
Inflorescence	60	0.83	0.88	0.86	‐6.02
Inflorescence	61	0.54	0.54	0.54	1.63
Inflorescence	62	0.90	0.93	0.91	‐3.15
Inflorescence	63	1.63	1.47	1.55	10.02
Resin	64	5.55	5.13	5.34	8.00
Resin	65	12.11	11.26	11.69	7.27
Resin	66	13.58	12.59	13.09	7.53
Inflorescence	67	3.34	3.31	3.33	0.94
Inflorescence	68	2.94	2.72	2.83	7.60
Inflorescence	69	3.77	3.61	3.69	4.34
Inflorescence	70	2.86	2.70	2.78	5.83
Resin	71	13.72	12.73	13.22	7.49
Inflorescence	72	1.18	1.27	1.23	‐7.54
Inflorescence	73	3.99	3.73	3.86	6.59
Inflorescence	74	5.41	4.96	5.18	8.80
Inflorescence	75	3.19	3.23	3.21	‐1.43
Inflorescence	76	5.41	4.93	5.17	9.23
Inflorescence	77	2.34	2.48	2.41	‐5.91
Inflorescence	78	3.28	3.21	3.25	2.10
Inflorescence	79	2.23	2.16	2.20	3.33
Inflorescence	80	0.95	0.98	0.96	‐2.97
Inflorescence	81	5.34	5.07	5.21	5.29
Inflorescence	82	5.73	5.77	5.75	‐0.72
Inflorescence	83	5.73	5.78	5.76	‐0.97
Inflorescence	84	–	–	–	–
Inflorescence	85	1.58	1.53	1.56	3.39
Inflorescence	86	0.72	0.66	0.69	7.90
Inflorescence	87	2.56	2.64	2.60	‐3.10
Inflorescence	88	0.96	0.85	0.91	11.68
Inflorescence	89	0.63	0.57	0.60	8.42
Inflorescence	90	–	–	–	–
Inflorescence	91	0.69	0.62	0.65	11.09
Inflorescence	92	0.73	0.75	0.74	‐2.95
Bias (%)	‐1.87				
Stand. Dev. (%) uLoA (%) lLoA (%)	6.51 10.90 ‐14.64				

Sample no, number of sample; Conc UHPLC, THC concentration obtained by UHPLC; Conc UHPSFC, THC concentration obtained by UHPSFC; True value, mean between Conc UHPLC and Conc UHPSFC values; Relative difference, relative difference between Conc UHPLC and Conc UHPSFC; Bias, average of relative difference percentages; Stand. Dev., standard deviation of relative difference percentages; LoA (+), positive limit of agreement; LoA (‐), negative limit of agreement.

F is a factor of 3.3 and 10 for LLOD and LLOQ, respectively;

SD is the residual standard deviation of the linear regression;

B is the slope of the regression line.

Concerning the UHPLC method, the estimated LLOD and LLOQ for CBD were 0.33 and 0.99 µg/mL, respectively, while for the UHPSFC method, 2.30 and 6.96 µg/mL, respectively. For the second analyte taken into consideration, CBN, the LLOD and LLOQ were 0.21 and 0.63 µg/mL, respectively, with the UHPLC method and 1.50 and 4.55 µg/mL, respectively, for the UHPSFC method. LLOD and LLOQ were also calculated for the other analytes quantified and are reported in Table S3. Although these limits present interesting values for both techniques, a relevant difference in terms of sensitivity has been highlighted for the PDA detector depending on the technique it was combined with. Other works have already noted and discussed this lack of sensitivity when a PDA detector is used in SFC with respect to when it is coupled with an LC system.^26^ The reason behind this lack of performance may be explained by the differences in solvating power and the refractive index of CO_2_, which can vary considerably, leading to a greater baseline noise.^27^ This aspect should be considered when applying SFC to limit assays for impurities in pharmaceuticals, for instance. In a cannabis analysis context, the only limit assay of interest could be that for the total THC amount (sum of THC and THCA‐A concentrations), which, according to the legislations of many countries, has to be less than 0.2‐1% w/w to not avoid classification of a cannabis sample as an illicit drug. Due to these low concentrations, MS detection is recommended to gain sensitivity.

The Bland‐Altman method was then applied as a statistical method to assess the degree of agreement between the quantitative measurements obtained by UHPSFC‐UV with respect to those obtained by UHPLC‐UV. Briefly, a Bland‐Altman plot, also called a difference plot, allows for highlighting of the differences in quantitative measurements executed with two analytical methods and the relationship between these differences and what is assumed to be the true value. This plot can be obtained in different ways depending on what is chosen to be plotted on the two axes.^28^ In this specific case, the authors chose to use the relative difference percentages because it is convenient when the methods show variability linked to increased magnitude. On the horizontal axis, the best estimate of the true value (the mean of the pair of measurements) was plotted. Concerning the construction of the limits of agreement (LoA), they are defined by calculating the mean and the standard deviation of the differences between the measurements. They are based on the assumption that the differences of measurements follow a Gaussian distribution, 95% of them being within these limits. The LoA include both systematic (bias) and random error (precision) and provide a useful measure for comparing the likely differences between individual results measured by two analytical methods.^29^

**FIGURE 4:**
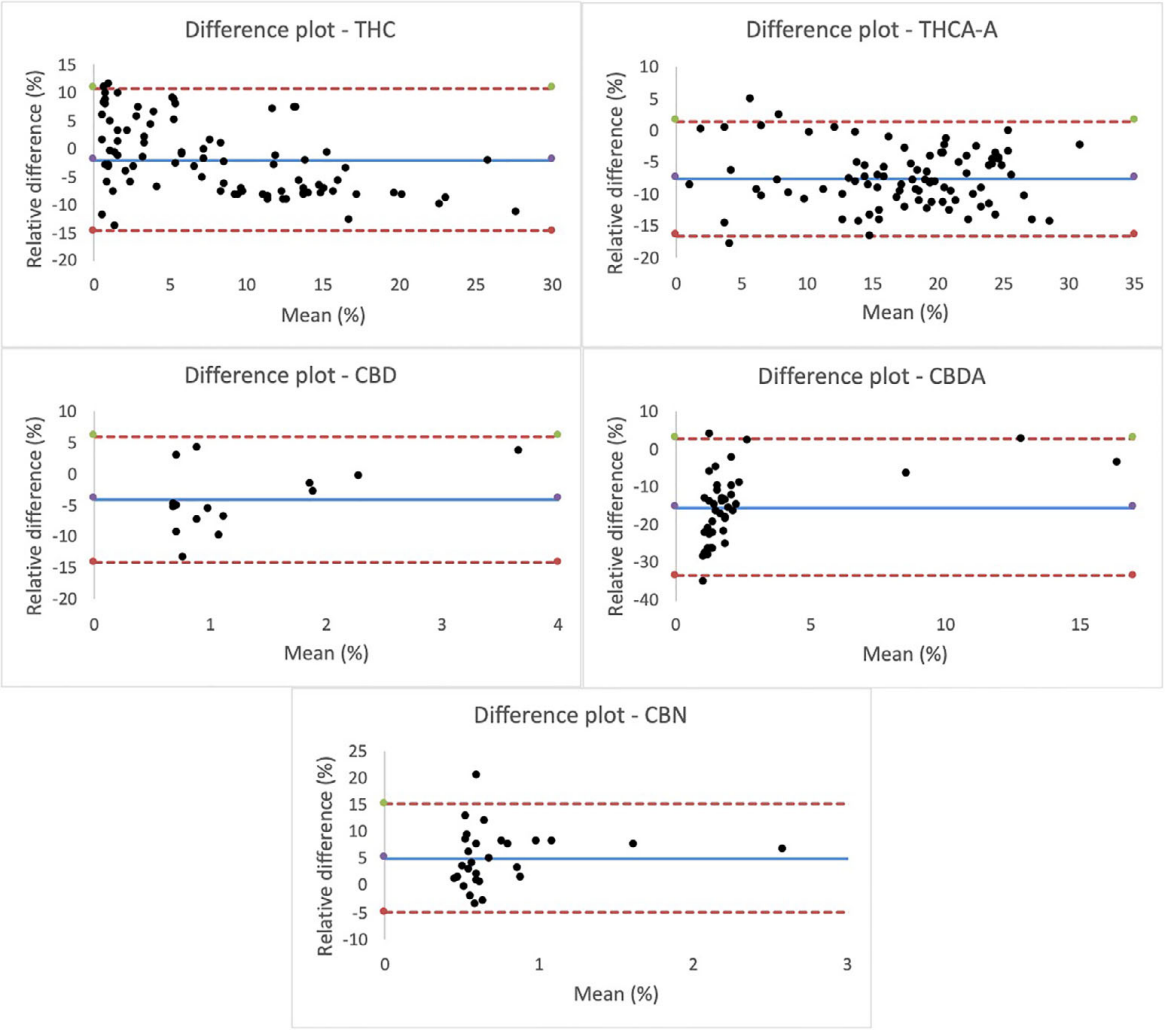
Bland‐Altman plots built on the basis of the quantitative results obtained for the following analytes: THC, THCA‐A, CBD, CBDA, and CBN

Figure 4 shows the Bland‐Altman plots calculated for the following analytes: (a) THC; (b) THCA‐A; (c) CBD; (d) CBDA; and (e) CBN. As mentioned above, they have been obtained by plotting the relative difference percentages on the vertical axis versus the mean of the values obtained by both techniques. The dotted red lines correspond to the upper and lower limits of agreement (LoA = ± bias ‐ 1.96 standard deviation), while the solid line corresponds to the bias. The latter gives a first idea about the agreement between the two methods. Indeed, it is calculated by the mean of the differences, which ideally should be zero. When the mean of differences is not zero, it means that there is an over‐ or underestimation equal to this mean for one method in respect to the supposed true values (which remain unknown). In the case of THC, one can notice that this error is –1.87 units of relative difference. This means that, on average, the UHPSFC method overestimates this analyte by 1.87 (units of relative differences). The same behavior was observed for THCA‐A, CBD, and CBDA. In contrast, CBN shows a bias value of 5.23, indicating an underestimation of this analyte when analyzed by UHPSFC. Looking at the results obtained for CBD, all samples presented differences within the established LoA. However, as seen in Figure 4, the following samples presented difference values that go beyond the LoA for the other analytes: for THC, samples n°88 (0.96% was determined via UHPLC vs 0.85% via UHPSFC) and n°91 (0.69% vs 0.62%); for THCA‐A, samples n°9 (5.71% vs 5.44%), n°12 (7.98% vs 7.79%), and n°72 (3.67% vs 4.39%); for CBD, samples n°17 (0.86% vs 1.22%) and n°34 (1.28% vs 1.23%); and for CBN, only sample n°12 (0.65% vs 0.53%). Despite the existence of these differences in terms of final concentration, they remain acceptable, and it can be stated that the quantitative results obtained with both techniques are strongly correlated, and the UHPSFC method, in most cases, perfectly agrees with the UHPLC method.

## 4 CONCLUSIONS

This work demonstrates the potential of UHPSFC in the context of cannabis analysis. UHPSFC, employing a mobile phase in super/subcritical conditions, allows for chromatographic analysis to be performed with a very short analysis time (6 min vs the 18 min of the reference UHPLC method used for this study). However, these particular conditions of the mobile phase demand precautions regarding the control of some CMPs, which can directly affect the qualitative chromatographic performance, highlighting once again the importance of the regular maintenance and qualification of the equipment. The selected final conditions allow for appropriate selectivity to potentially quantify nine phyto‐cannabinoids employing a low amount of organic solvent. The simulated routine use performed on a very large quantity of real cannabis samples allowed for study of the degree of accordance between the results generated by UHPSFC and those obtained with the gold‐standard technique used for cannabinoid analysis, UHPLC. The quantitative performance of UHPSFC was in accordance with the reference technique, confirming the suitability of this technique in the context of cannabinoid analysis. During this simulation, some issues that could be encountered when analyzing cannabis samples have arisen, such as the selectivity problem discussed above (Section 3.3). Another limitation related to UHPSFC‐UV is the lower sensitivity if compared to UHPLC‐UV, which must be considered when approaching minor compounds and during sample preparation. In conclusion, SFC is an interesting alternative in the analytical chemist's toolbox for cannabis testing. In particular, for those applications that involve a large number of samples and require a rapid result such as the cannabis screening of police seizures.

## CONFLICT OF INTEREST

The authors declare no conflict of interest.

## Supporting information

Supporting information
