# Supplementary material for: The analysis of cannabinoids in cannabis samples by supercritical fluid chromatography and ultra‐high‐performance liquid chromatography: A comparison study
Source: Anal Sci Adv. 2020 Nov 26;2(1-2):2–14. doi: 10.1002/ansa.202000091 (PMC10989068; doi:10.1002/ansa.202000091)
Supplement: Supplementary file 1 — Supporting information [file ANSA-2-2-s001.docx]

**SUPPLEMENTARY MATERIAL**

**The analysis of cannabinoids in cannabis samples by supercritical fluid chromatography and ultra-high performance liquid chromatography: a comparison study**

Riccardo Deidda^a*^, Cédric Schelling^b^, Jean-Marc Roussel^c^, Amandine Dispas^a,d^, Charlotte De Bleye^a^, Éric Ziemons^a^, Philippe Hubert^a^, Jean-Luc Veuthey^b^

*^a^University of Liège (ULiège), CIRM, Laboratory of Pharmaceutical Analytical Chemistry, B36 Tower 4 Avenue Hippocrate 15, 4000 Liège, Belgium*

*^b^University of Geneva, School of Pharmaceutical Sciences, rue Michel Servet 1, 1211 Geneva 4, Switzerland*

*^c^Consultant, 389 Quai Jean Jaurès, 71000 Mâcon, France*

*^d^University of Liège, CIRM, Laboratory of Medicine Analysis, B36 Tower 4 Avenue Hippocrate 15, 4000 Liège, Belgium*

*Corresponding author. *E-mail address:* [riccardo.deidda@uliege.be](mailto:riccardo.deidda@uliege.be) (R. Deidda); Tel.: +32 (0) 4 366 4319; Fax: +32 (0) 4 366 4317

**Tab. S1.** Robustness results. Color codes: light green, non-significant; light yellow, weakly significant; light orange, significant; light red, highly significant.

1. Effects on *R_sCBD-Δ8-THC_*

Mean value at the center of design space experimental results: 2.135; b0 effect: 2.264.

| Factor / Interaction | Effect | Stat. Signif. (p value %) | Calc. response at level  -1 | Calc. response at level  +1 | Robustness specification | CONCLUSION |
| --- | --- | --- | --- | --- | --- | --- |
| X1:HCOOH | -0.0117 | 13.3 | 2.276 | 2.252 | Rs >1.5 | Robust |
| X2: Temp | 0.1021 | < 0.01 | 2.162 | 2.366 | Rs >1.5 | Robust |
| X3: Col | -0.1591 | < 0.01 | 2.423 | 2.105 | Rs >1.5 | Robust |
| X4: Grad | -0.2772 | < 0.01 | 2.541 | 1.987 | Rs >1.5 | Robust |
| X1X2 | -0.0059 | 40.5 | 2.270 | 2.258 | Rs >1.5 | Robust |
| X1X3 | 0.0017 | 80.6 | 2.262 | 2.266 | Rs >1.5 | Robust |
| X2X3 | 0.0112 | 14.7 | 2.253 | 2.275 | Rs >1.5 | Robust |
| X1X4 | -0.0014 | 83.4 | 2.265 | 2.263 | Rs >1.5 | Robust |
| X2X4 | 0.0188 | 3.45 | 2.245 | 2.283 | Rs >1.5 | Robust |
| X3X4 | 0.0237 | 1.51 | 2.241 | 2.288 | Rs >1.5 | Robust |

1. Effects on *R_sΔ8-THC-THC_*

Mean value at the center of design space experimental results: 1.804; b0 effect: 1.808.

| Factor / Interaction | Effect | Stat. Signif. (p value %) | Calc. response at level  -1 | Calc. response at level  +1 | Robustness specification | CONCLUSION |
| --- | --- | --- | --- | --- | --- | --- |
| X1: HCOOH | -0.0101 | 7.6 | 1.818 | 1.798 | Rs >1.5 | Robust |
| X2: Temp | -0.0134 | 3.19 | 1.821 | 1.795 | Rs >1.5 | Robust |
| X3: Col | 0.0229 | 0.395 | 1.785 | 1.831 | Rs >1.5 | Robust |
| X4: Grad | -0.1058 | < 0.01 | 1.913 | 1.702 | Rs >1.5 | Robust |
| X1X2 | 0.0040 | 41.8 | 1.804 | 1.812 | Rs >1.5 | Robust |
| X1X3 | -0.0002 | 95.8 | 1.808 | 1.808 | Rs >1.5 | Robust |
| X2X3 | 0.0110 | 6.0 | 1.797 | 1.819 | Rs >1.5 | Robust |
| X1X4 | -0.0021 | 65.9 | 1.810 | 1.806 | Rs >1.5 | Robust |
| X2X4 | 0.0121 | 4.41 | 1.796 | 1.820 | Rs >1.5 | Robust |
| X3X4 | -0.0044 | 37.9 | 1.812 | 1.804 | Rs >1.5 | Robust |

1. Effects on *R_sTHC-CBC_*

Mean value at the center of design space experimental results: 2.661; b0 effect: 2.875.

| Factor / Interaction | Effect | Stat. Signif. (p value %) | Calc. response at level  -1 | Calc. response at level  +1 | Robustness specification | CONCLUSION |
| --- | --- | --- | --- | --- | --- | --- |
| X1: HCOOH | 0.0034 | 78.1 | 2.872 | 2.878 | Rs >1.5 | Robust |
| X2: Temp | 0.2361 | < 0.01 | 2.639 | 3.111 | Rs >1.5 | Robust |
| X3: Col | -0.2764 | < 0.01 | 3.151 | 2.599 | Rs >1.5 | Robust |
| X4: Grad | -0.2799 | < 0.01 | 3.155 | 2.595 | Rs >1.5 | Robust |
| X1X2 | -0.0094 | 45.7 | 2.884 | 2.866 | Rs >1.5 | Robust |
| X1X3 | -0.0019 | 87.5 | 2.877 | 2.873 | Rs >1.5 | Robust |
| X2X3 | 0.0134 | 30.3 | 2.862 | 2.888 | Rs >1.5 | Robust |
| X1X4 | 0.0036 | 77.3 | 2.871 | 2.879 | Rs >1.5 | Robust |
| X2X4 | -0.0043 | 72.8 | 2.879 | 2.871 | Rs >1.5 | Robust |
| X3X4 | 0.0422 | 1.54 | 2.833 | 2.917 | Rs >1.5 | Robust |

1. Effects on *R_sCBN – THCA–A_*

Mean value at the center of design space experimental results: 2.438; b0 effect: 2.594.

| Factor / Interaction | Effect | Stat. Signif. (p value %) | Calc. response at level  -1 | Calc. response at level  +1 | Robustness specification | CONCLUSION |
| --- | --- | --- | --- | --- | --- | --- |
| X1: HCOOH | -0.721 | < 0.01 | 3.315 | 1.873 | Rs >1.5 | Robust |
| X2: Temp | 1.277 | < 0.01 | 1.317 | 3.871 | Rs >1.5 | Not Robust |
| X3: Col | -0.144 | 0.217 | 2.738 | 2.450 | Rs >1.5 | Robust |
| X4: Grad | -0.303 | < 0.01 | 2.897 | 2.291 | Rs >1.5 | Robust |
| X1X2 | 0.042 | 15.6 | 2.552 | 2.636 | Rs >1.5 | Robust |
| X1X3 | -0.020 | 46.0 | 2.614 | 2.574 | Rs >1.5 | Robust |
| X2X3 | 0.005 | 85.1 | 2.589 | 2.599 | Rs >1.5 | Robust |
| X1X4 | -0.041 | 15.8 | 2.635 | 2.553 | Rs >1.5 | Robust |
| X2X4 | 0.020 | 45.7 | 2.574 | 2.614 | Rs >1.5 | Robust |
| X3X4 | 0.070 | 3.71 | 2.524 | 2.664 | Rs >1.5 | Robust |

1. Effects on *R_sCBDA – CBG_*

Mean value at the center of design space experimental results: 2.542; b0 effect: 2.502.

| Factor / Interaction | Effect | Stat. Signif. (p value %) | Calc. response at level  -1 | Calc. response at level  +1 | Robustness specification | CONCLUSION |
| --- | --- | --- | --- | --- | --- | --- |
| X1: HCOOH | 0.711 | < 0.01 | 1.791 | 3.213 | Rs >1.5 | Robust |
| X2: Temp | -1.438 | < 0.01 | 3.940 | 1.064 | Rs >1.5 | Not Robust |
| X3: Col | -0.169 | 0.0106 | 2.671 | 2.333 | Rs >1.5 | Robust |
| X4: Grad | 0.210 | < 0.01 | 2.292 | 2.712 | Rs >1.5 | Robust |
| X1X2 | 0.098 | 0.138 | 2.404 | 2.600 | Rs >1.5 | Robust |
| X1X3 | -0.004 | 82.9 | 2.506 | 2.498 | Rs >1.5 | Robust |
| X2X3 | 0.028 | 12.9 | 2.474 | 2.530 | Rs >1.5 | Robust |
| X1X4 | 0.001 | 95.1 | 2.501 | 2.503 | Rs >1.5 | Robust |
| X2X4 | 0.030 | 11.1 | 2.472 | 2.532 | Rs >1.5 | Robust |
| X3X4 | -0.078 | 0.39 | 2.580 | 2.424 | Rs >1.5 | Robust |

**Tab. S2.** Quantitative results for: b) THCA-A; c) CBD; d) CBDA; e) CBN.

b) Quantitative data for THCA-A used to evaluate the agreement between the two analytical methods, UHPLC and UHPSFC. The table includes also the true values, the relative difference percentages, the bias and the values of the limits of agreement used to obtain the Bland-Altman plot.

| Cannabis sample type | Sample no | Conc UHPLC  (% w/w) | Conc UHPSFC  (% w/w) | True value (% w/w) | Relative difference percentage (%) |
| --- | --- | --- | --- | --- | --- |
| Resin | 1 | 25.26 | 29.05 | 27.16 | -13.95 |
| Resin | 2 | 13.84 | 14.90 | 14.37 | -7.40 |
| Resin | 3 | 15.38 | 16.28 | 15.83 | -5.69 |
| Resin | 4 | 5.87 | 6.44 | 6.16 | -9.36 |
| Resin | 5 | - | - | - | - |
| Resin | 6 | 15.91 | 17.66 | 16.79 | -10.44 |
| Resin | 7 | 25.22 | 27.96 | 26.59 | -10.33 |
| Resin | 8 | 18.43 | 20.61 | 19.52 | -11.17 |
| Resin | 9 | 5.71 | 5.44 | 5.58 | 4.84 |
| Resin | 10 | 20.20 | 22.53 | 21.36 | -10.94 |
| Resin | 11 | 16.22 | 17.84 | 17.03 | -9.48 |
| Resin | 12 | 7.98 | 7.79 | 7.88 | 2.42 |
| Resin | 13 | 10.15 | 10.18 | 10.16 | -0.32 |
| Resin | 14 | 20.00 | 20.72 | 20.36 | -3.53 |
| Resin | 15 | 25.36 | 25.37 | 25.37 | -0.04 |
| Resin | 16 | 22.51 | 25.24 | 23.88 | -11.45 |
| Resin | 17 | 14.44 | 16.63 | 15.54 | -14.10 |
| Resin | 18 | 21.89 | 24.67 | 23.28 | -11.95 |
| Resin | 19 | 24.08 | 25.15 | 24.61 | -4.33 |
| Resin | 20 | 22.75 | 26.01 | 24.38 | -13.37 |
| Resin | 21 | 22.67 | 23.27 | 22.97 | -2.62 |
| Resin | 22 | 17.21 | 17.70 | 17.45 | -2.80 |
| Resin | 23 | 24.96 | 25.82 | 25.39 | -3.39 |
| Resin | 24 | 23.47 | 24.74 | 24.11 | -5.28 |
| Resin | 25 | 19.84 | 20.56 | 20.20 | -3.59 |
| Resin | 26 | 16.36 | 18.47 | 17.41 | -12.08 |
| Resin | 27 | 23.96 | 24.84 | 24.40 | -3.60 |
| Resin | 28 | 23.55 | 24.64 | 24.10 | -4.49 |
| Resin | 29 | 20.30 | 20.75 | 20.53 | -2.19 |
| Resin | 30 | 20.49 | 20.77 | 20.63 | -1.33 |
| Resin | 31 | 17.55 | 19.58 | 18.56 | -10.95 |
| Resin | 32 | 20.02 | 22.03 | 21.03 | -9.58 |
| Resin | 33 | 13.67 | 13.71 | 13.69 | -0.23 |
| Resin | 34 | 14.56 | 16.50 | 15.53 | -12.54 |
| Resin | 35 | 18.98 | 19.74 | 19.36 | -3.95 |
| Resin | 36 | 26.42 | 30.50 | 28.46 | -14.30 |
| Resin | 37 | 19.24 | 21.53 | 20.39 | -11.22 |
| Resin | 38 | 20.78 | 23.88 | 22.33 | -13.91 |
| Resin | 39 | 1.88 | 1.88 | 1.88 | 0.08 |
| Resin | 40 | 18.00 | 20.35 | 19.17 | -12.28 |
| Resin | 41 | 19.58 | 22.21 | 20.89 | -12.57 |
| Resin | 42 | 20.03 | 20.76 | 20.40 | -3.58 |
| Resin | 43 | 24.22 | 25.62 | 24.92 | -5.60 |
| Resin | 44 | 30.47 | 31.14 | 30.81 | -2.18 |
| Inflorescence | 45 | 17.41 | 19.09 | 18.25 | -9.20 |
| Inflorescence | 46 | 6.51 | 6.46 | 6.49 | 0.72 |
| Inflorescence | 47 | 12.71 | 13.72 | 13.22 | -7.61 |
| Inflorescence | 48 | 14.02 | 15.29 | 14.66 | -8.63 |
| Inflorescence | 49 | 16.42 | 17.89 | 17.15 | -8.58 |
| Inflorescence | 50 | 14.62 | 16.02 | 15.32 | -9.14 |
| Inflorescence | 51 | 17.61 | 19.36 | 18.48 | -9.45 |
| Inflorescence | 52 | 12.03 | 13.30 | 12.67 | -10.06 |
| Inflorescence | 53 | 19.02 | 20.60 | 19.81 | -7.96 |
| Inflorescence | 54 | 21.75 | 22.64 | 22.19 | -3.97 |
| Inflorescence | 55 | 12.06 | 12.00 | 12.03 | 0.44 |
| Inflorescence | 56 | - | - | - | - |
| Inflorescence | 57 | - | - | - | - |
| Inflorescence | 58 | 21.07 | 22.14 | 21.60 | -4.97 |
| Inflorescence | 59 | 18.77 | 20.36 | 19.56 | -8.14 |
| Inflorescence | 60 | 21.45 | 22.95 | 22.20 | -6.75 |
| Inflorescence | 61 | 13.40 | 14.10 | 13.75 | -5.10 |
| Inflorescence | 62 | 17.49 | 18.44 | 17.96 | -5.29 |
| Inflorescence | 63 | 13.12 | 14.21 | 13.67 | -7.97 |
| Resin | 64 | 13.78 | 15.74 | 14.76 | -13.24 |
| Resin | 65 | 0.95 | 1.04 | 0.99 | -8.65 |
| Resin | 66 | 13.97 | 14.77 | 14.37 | -5.53 |
| Inflorescence | 67 | 14.78 | 15.87 | 15.32 | -7.07 |
| Inflorescence | 68 | 6.16 | 6.83 | 6.50 | -10.25 |
| Inflorescence | 69 | 10.67 | 11.70 | 11.19 | -9.22 |
| Inflorescence | 70 | 4.01 | 4.27 | 4.14 | -6.28 |
| Resin | 71 | 8.12 | 8.94 | 8.53 | -9.66 |
| Inflorescence | 72 | 3.67 | 4.39 | 4.03 | -17.78 |
| Inflorescence | 73 | 3.40 | 3.94 | 3.67 | -14.63 |
| Inflorescence | 74 | 7.38 | 7.99 | 7.69 | -7.88 |
| Inflorescence | 75 | 12.89 | 14.85 | 13.87 | -14.16 |
| Inflorescence | 76 | 15.28 | 16.45 | 15.86 | -7.35 |
| Inflorescence | 77 | 11.82 | 13.60 | 12.71 | -13.95 |
| Inflorescence | 78 | 13.51 | 15.93 | 14.72 | -16.41 |
| Inflorescence | 79 | 9.25 | 10.29 | 9.77 | -10.71 |
| Inflorescence | 80 | 18.61 | 20.20 | 19.41 | -8.24 |
| Inflorescence | 81 | 16.18 | 16.36 | 16.27 | -1.08 |
| Inflorescence | 82 | 17.37 | 18.76 | 18.06 | -7.70 |
| Inflorescence | 83 | 17.80 | 18.94 | 18.37 | -6.18 |
| Inflorescence | 84 | 24.72 | 26.53 | 25.63 | -7.06 |
| Inflorescence | 85 | 21.55 | 23.85 | 22.70 | -10.14 |
| Inflorescence | 86 | 18.24 | 19.70 | 18.97 | -7.72 |
| Inflorescence | 87 | 3.69 | 3.68 | 3.69 | 0.37 |
| Inflorescence | 88 | 19.61 | 21.44 | 20.52 | -8.91 |
| Inflorescence | 89 | 23.21 | 24.55 | 23.88 | -5.61 |
| Inflorescence | 90 | 24.07 | 25.21 | 24.64 | -4.63 |
| Inflorescence | 91 | 22.28 | 24.39 | 23.33 | -9.06 |
| Inflorescence | 92 | 18.51 | 19.74 | 19.12 | -6.44 |
| Bias (%) | -7.43 |  |  |  |  |
| Stand.Dev.(%)  uLoA (%)  lLoA (%) | 4.58  1.54  -16.41 |  |  |  |  |
|  |  |  |  |  |  |

c) Quantitative data for CBD used to evaluate the agreement between the two analytical methods, UHPLC and UHPSFC. The table includes also the true values, the relative difference percentages, the bias and the values of the limits of agreement used to obtain the Bland-Altman plot.

| Cannabis sample type | Sample no | Conc UHPLC  (% w/w) | Conc UHPSFC  (% w/w) | True value (% w/w) | Relative difference percentage (%) |
| --- | --- | --- | --- | --- | --- |
| Resin | 1 | - | - | - | - |
| Resin | 2 | 1.07 | 1.15 | 1.11 | -6.70 |
| Resin | 3 | - | - | - | - |
| Resin | 4 | - | - | - | - |
| Resin | 5 | - | - | - | - |
| Resin | 6 | - | - | - | - |
| Resin | 7 | - | - | - | - |
| Resin | 8 | - | - | - | - |
| Resin | 9 | - | - | - | - |
| Resin | 10 | - | - | - | - |
| Resin | 11 | - | - | - | - |
| Resin | 12 | - | - | - | - |
| Resin | 13 | - | - | - | - |
| Resin | 14 | - | - | - | - |
| Resin | 15 | - | - | - | - |
| Resin | 16 | - | - | - | - |
| Resin | 17 | - | - | - | - |
| Resin | 18 | - | - | - | - |
| Resin | 19 | - | - | - | - |
| Resin | 20 | - | - | - | - |
| Resin | 21 | - | - | - | - |
| Resin | 22 | 1.02 | 1.13 | 1.07 | -9.78 |
| Resin | 23 | - | - | - | - |
| Resin | 24 | - | - | - | - |
| Resin | 25 | 0.66 | 0.69 | 0.68 | -4.83 |
| Resin | 26 | - | - | - | - |
| Resin | 27 | - | - | - | - |
| Resin | 28 | - | - | - | - |
| Resin | 29 | 0.68 | 0.74 | 0.71 | -9.33 |
| Resin | 30 | - | - | - | - |
| Resin | 31 | - | - | - | - |
| Resin | 32 | - | - | - | - |
| Resin | 33 | 0.66 | 0.70 | 0.68 | -5.17 |
| Resin | 34 | 0.85 | 0.91 | 0.88 | -7.38 |
| Resin | 35 | 0.72 | 0.82 | 0.77 | -13.22 |
| Resin | 36 | - | - | - | - |
| Resin | 37 | - | - | - | - |
| Resin | 38 | - | - | - | - |
| Resin | 39 | - | - | - | - |
| Resin | 40 | - | - | - | - |
| Resin | 41 | - | - | - | - |
| Resin | 42 | - | - | - | - |
| Resin | 43 | - | - | - | - |
| Resin | 44 | - | - | - | - |
| Inflorescence | 45 | - | - | - | - |
| Inflorescence | 46 | - | - | - | - |
| Inflorescence | 47 | - | - | - | - |
| Inflorescence | 48 | - | - | - | - |
| Inflorescence | 49 | - | - | - | - |
| Inflorescence | 50 | - | - | - | - |
| Inflorescence | 51 | - | - | - | - |
| Inflorescence | 52 | - | - | - | - |
| Inflorescence | 53 | - | - | - | - |
| Inflorescence | 54 | - | - | - | - |
| Inflorescence | 55 | 0.69 | 0.73 | 0.71 | -5.05 |
| Inflorescence | 56 | 2.27 | 2.28 | 2.28 | -0.35 |
| Inflorescence | 57 | 1.86 | 1.91 | 1.88 | -2.68 |
| Inflorescence | 58 | - | - | - | - |
| Inflorescence | 59 | - | - | - | - |
| Inflorescence | 60 | - | - | - | - |
| Inflorescence | 61 | - | - | - | - |
| Inflorescence | 62 | - | - | - | - |
| Inflorescence | 63 | - | - | - | - |
| Resin | 64 | 0.96 | 1.01 | 0.98 | -5.43 |
| Resin | 65 | 3.72 | 3.59 | 3.66 | 3.69 |
| Resin | 66 | 0.90 | 0.87 | 0.88 | 4.29 |
| Inflorescence | 67 | - | - | - | - |
| Inflorescence | 68 | - | - | - | - |
| Inflorescence | 69 | - | - | - | - |
| Inflorescence | 70 | - | - | - | - |
| Resin | 71 | 0.71 | 0.69 | 0.70 | 3.06 |
| Inflorescence | 72 | - | - | - | - |
| Inflorescence | 73 | - | - | - | - |
| Inflorescence | 74 | - | - | - | - |
| Inflorescence | 75 | - | - | - | - |
| Inflorescence | 76 | - | - | - | - |
| Inflorescence | 77 | - | - | - | - |
| Inflorescence | 78 | - | - | - | - |
| Inflorescence | 79 | - | - | - | - |
| Inflorescence | 80 | - | - | - | - |
| Inflorescence | 81 | - | - | - | - |
| Inflorescence | 82 | - | - | - | - |
| Inflorescence | 83 | - | - | - | - |
| Inflorescence | 84 | - | - | - | - |
| Inflorescence | 85 | - | - | - | - |
| Inflorescence | 86 | - | - | - | - |
| Inflorescence | 87 | 1.85 | 1.88 | 1.86 | -1.53 |
| Inflorescence | 88 | - | - | - | - |
| Inflorescence | 89 | - | - | - | - |
| Inflorescence | 90 | - | - | - | - |
| Inflorescence | 91 | - | - | - | - |
| Inflorescence | 92 | - | - | - | - |
| Bias (%) | -4.03 |  |  |  |  |
| Stand.Dev.(%)  uLoA (%)  lLoA (%) | 5.14  6.04  -14.10 |  |  |  |  |
|  |  |  |  |  |  |

d) Quantitative data for CBDA used to evaluate the agreement between the two analytical methods, UHPLC and UHPSFC. The table includes also the true values, the relative difference percentages, the bias and the values of the limits of agreement used to obtain the Bland-Altman plot.

| Cannabis sample type | Sample no | Conc UHPLC  (% w/w) | Conc UHPSFC  (% w/w) | True value (% w/w) | Relative difference percentage (%) |
| --- | --- | --- | --- | --- | --- |
| Resin | 1 | 1.74 | 1.98 | 1.86 | -13.17 |
| Resin | 2 | 1.65 | 1.99 | 1.82 | -18.51 |
| Resin | 3 | 0.99 | 1.13 | 1.06 | -13.12 |
| Resin | 4 | - | - | - | - |
| Resin | 5 | - | - | - | - |
| Resin | 6 | 1.19 | 1.37 | 1.28 | -13.76 |
| Resin | 7 | 1.61 | 1.84 | 1.72 | -13.75 |
| Resin | 8 | - | - | - | - |
| Resin | 9 | 1.19 | 1.26 | 1.22 | -5.75 |
| Resin | 10 | 1.37 | 1.61 | 1.49 | -16.08 |
| Resin | 11 | - | - | - | - |
| Resin | 12 | - | - | - | - |
| Resin | 13 | - | - | - | - |
| Resin | 14 | 1.21 | 1.58 | 1.39 | -26.37 |
| Resin | 15 | 1.46 | 1.60 | 1.53 | -9.57 |
| Resin | 16 | 0.92 | 1.22 | 1.07 | -27.47 |
| Resin | 17 | 0.86 | 1.22 | 1.04 | -34.90 |
| Resin | 18 | 0.88 | 1.18 | 1.03 | -28.22 |
| Resin | 19 | 1.80 | 2.10 | 1.95 | -15.55 |
| Resin | 20 | 1.16 | 1.51 | 1.34 | -26.22 |
| Resin | 21 | 1.33 | 1.54 | 1.44 | -14.46 |
| Resin | 22 | 2.25 | 2.46 | 2.36 | -8.83 |
| Resin | 23 | 1.66 | 1.98 | 1.82 | -17.75 |
| Resin | 24 | 1.22 | 1.52 | 1.37 | -21.96 |
| Resin | 25 | 1.93 | 2.27 | 2.10 | -16.23 |
| Resin | 26 | - | - | - | - |
| Resin | 27 | 1.58 | 2.03 | 1.81 | -25.12 |
| Resin | 28 | 1.52 | 1.80 | 1.66 | -16.91 |
| Resin | 29 | 1.95 | 2.20 | 2.08 | -12.00 |
| Resin | 30 | 1.07 | 1.31 | 1.19 | -20.65 |
| Resin | 31 | 1.48 | 1.65 | 1.57 | -10.64 |
| Resin | 32 | - | - | - | - |
| Resin | 33 | 1.26 | 1.53 | 1.39 | -19.24 |
| Resin | 34 | 1.28 | 1.23 | 1.26 | 4.32 |
| Resin | 35 | 1.96 | 2.16 | 2.06 | -9.64 |
| Resin | 36 | 1.61 | 2.00 | 1.80 | -21.75 |
| Resin | 37 | - | - | - | - |
| Resin | 38 | 1.10 | 1.38 | 1.24 | -22.29 |
| Resin | 39 | - | - | - | - |
| Resin | 40 | - | - | - | - |
| Resin | 41 | 1.02 | 1.35 | 1.18 | -27.77 |
| Resin | 42 | 1.02 | 1.33 | 1.18 | -26.35 |
| Resin | 43 | 1.63 | 1.86 | 1.75 | -13.09 |
| Resin | 44 | 2.06 | 2.39 | 2.22 | -14.54 |
| Inflorescence | 45 | - | - | - | - |
| Inflorescence | 46 | - | - | - | - |
| Inflorescence | 47 | - | - | - | - |
| Inflorescence | 48 | - | - | - | - |
| Inflorescence | 49 | - | - | - | - |
| Inflorescence | 50 | - | - | - | - |
| Inflorescence | 51 | - | - | - | - |
| Inflorescence | 52 | - | - | - | - |
| Inflorescence | 53 | - | - | - | - |
| Inflorescence | 54 | - | - | - | - |
| Inflorescence | 55 | 2.06 | 2.10 | 2.08 | -1.95 |
| Inflorescence | 56 | 16.08 | 16.62 | 16.35 | -3.30 |
| Inflorescence | 57 | 12.99 | 12.65 | 12.82 | 2.69 |
| Inflorescence | 58 | - | - | - | - |
| Inflorescence | 59 | - | - | - | - |
| Inflorescence | 60 | - | - | - | - |
| Inflorescence | 61 | - | - | - | - |
| Inflorescence | 62 | - | - | - | - |
| Inflorescence | 63 | - | - | - | - |
| Resin | 64 | 2.70 | 2.63 | 2.66 | 2.43 |
| Resin | 65 | 0.93 | 1.16 | 1.05 | -21.86 |
| Resin | 66 | 1.47 | 1.54 | 1.51 | -4.60 |
| Inflorescence | 67 | - | - | - | - |
| Inflorescence | 68 | - | - | - | - |
| Inflorescence | 69 | - | - | - | - |
| Inflorescence | 70 | - | - | - | - |
| Resin | 71 | - | - | - | - |
| Inflorescence | 72 | - | - | - | - |
| Inflorescence | 73 | - | - | - | - |
| Inflorescence | 74 | - | - | - | - |
| Inflorescence | 75 | - | - | - | - |
| Inflorescence | 76 | - | - | - | - |
| Inflorescence | 77 | - | - | - | - |
| Inflorescence | 78 | - | - | - | - |
| Inflorescence | 79 | - | - | - | - |
| Inflorescence | 80 | - | - | - | - |
| Inflorescence | 81 | - | - | - | - |
| Inflorescence | 82 | - | - | - | - |
| Inflorescence | 83 | - | - | - | - |
| Inflorescence | 84 | - | - | - | - |
| Inflorescence | 85 | - | - | - | - |
| Inflorescence | 86 | - | - | - | - |
| Inflorescence | 87 | 8.26 | 8.78 | 8.52 | -6.06 |
| Inflorescence | 88 | - | - | - | - |
| Inflorescence | 89 | - | - | - | - |
| Inflorescence | 90 | - | - | - | - |
| Inflorescence | 91 | - | - | - | - |
| Inflorescence | 92 | - | - | - | - |
| Bias (%) | -15.25 |  |  |  |  |
| Stand.Dev.(%)  uLoA (%)  lLoA (%) | 9.27  2.92  -33.42 |  |  |  |  |
|  |  |  |  |  |  |

e) Quantitative data for CBN used to evaluate the agreement between the two analytical methods, UHPLC and UHPSFC. The table includes also the true values, the relative difference percentages, the bias and the values of the limits of agreement used to obtain the Bland-Altman plot.

| Cannabis sample type | Sample no | Conc UHPLC  (% w/w) | Conc UHPSFC  (% w/w) | True value (% w/w) | Relative difference percentage (%) |
| --- | --- | --- | --- | --- | --- |
| Resin | 1 | - | - | - | - |
| Resin | 2 | - | - | - | - |
| Resin | 3 | - | - | - | - |
| Resin | 4 | - | - | - | - |
| Resin | 5 | 0.69 | 0.66 | 0.67 | 5.18 |
| Resin | 6 | 0.79 | 0.72 | 0.75 | 8.21 |
| Resin | 7 | - | - | - | - |
| Resin | 8 | - | - | - | - |
| Resin | 9 | 1.12 | 1.03 | 1.07 | 8.43 |
| Resin | 10 | - | - | - | - |
| Resin | 11 | - | - | - | - |
| Resin | 12 | 0.65 | 0.53 | 0.59 | 20.42 |
| Resin | 13 | 0.57 | 0.55 | 0.56 | 4.19 |
| Resin | 14 | - | - | - | - |
| Resin | 15 | - | - | - | - |
| Resin | 16 | 0.63 | 0.65 | 0.64 | -2.74 |
| Resin | 17 | 0.60 | 0.59 | 0.60 | 0.96 |
| Resin | 18 | 0.62 | 0.61 | 0.61 | 0.69 |
| Resin | 19 | - | - | - | - |
| Resin | 20 | 0.55 | 0.54 | 0.54 | 3.23 |
| Resin | 21 | 0.47 | 0.47 | 0.47 | 1.75 |
| Resin | 22 | 0.87 | 0.84 | 0.86 | 3.44 |
| Resin | 23 | - | - | - | - |
| Resin | 24 | 0.55 | 0.50 | 0.53 | 9.36 |
| Resin | 25 | 0.61 | 0.56 | 0.59 | 7.87 |
| Resin | 26 | - | - | - | - |
| Resin | 27 | - | - | - | - |
| Resin | 28 | 0.45 | 0.45 | 0.45 | 1.32 |
| Resin | 29 | 0.58 | 0.60 | 0.58 | -3.31 |
| Resin | 30 | - | - | - | - |
| Resin | 31 | - | - | - | - |
| Resin | 32 | 0.82 | 0.76 | 0.79 | 7.77 |
| Resin | 33 | 0.68 | 0.60 | 0.64 | 12.13 |
| Resin | 34 | 0.54 | 0.56 | 0.55 | -1.97 |
| Resin | 35 | 0.56 | 0.53 | 0.54 | 6.15 |
| Resin | 36 | - | - | - | - |
| Resin | 37 | 0.55 | 0.48 | 0.52 | 12.84 |
| Resin | 38 | 0.51 | 0.49 | 0.50 | 3.60 |
| Resin | 39 | 0.88 | 0.87 | 0.88 | 1.54 |
| Resin | 40 | - | - | - | - |
| Resin | 41 | 0.51 | 0.51 | 0.51 | -0.19 |
| Resin | 42 | 0.54 | 0.49 | 0.52 | 8.49 |
| Resin | 43 | - | - | - | - |
| Resin | 44 | - | - | - | - |
| Inflorescence | 45 | - | - | - | - |
| Inflorescence | 46 | - | - | - | - |
| Inflorescence | 47 | - | - | - | - |
| Inflorescence | 48 | - | - | - | - |
| Inflorescence | 49 | - | - | - | - |
| Inflorescence | 50 | - | - | - | - |
| Inflorescence | 51 | - | - | - | - |
| Inflorescence | 52 | - | - | - | - |
| Inflorescence | 53 | - | - | - | - |
| Inflorescence | 54 | - | - | - | - |
| Inflorescence | 55 | - | - | - | - |
| Inflorescence | 56 | - | - | - | - |
| Inflorescence | 57 | - | - | - | - |
| Inflorescence | 58 | - | - | - | - |
| Inflorescence | 59 | - | - | - | - |
| Inflorescence | 60 | - | - | - | - |
| Inflorescence | 61 | - | - | - | - |
| Inflorescence | 62 | - | - | - | - |
| Inflorescence | 63 | - | - | - | - |
| Resin | 64 | 1.02 | 0.94 | 0.98 | 8.38 |
| Resin | 65 | 2.67 | 2.49 | 2.58 | 6.89 |
| Resin | 66 | 1.67 | 1.55 | 1.61 | 7.67 |
| Inflorescence | 67 | - | - | - | - |
| Inflorescence | 68 | - | - | - | - |
| Inflorescence | 69 | - | - | - | - |
| Inflorescence | 70 | - | - | - | - |
| Resin | 71 | 3.32 | 3.09 | 3.20 | 7.37 |
| Inflorescence | 72 | - | - | - | - |
| Inflorescence | 73 | 0.60 | 0.59 | 0.59 | 2.11 |
| Inflorescence | 74 | - | - | - | - |
| Inflorescence | 75 | - | - | - | - |
| Inflorescence | 76 | - | - | - | - |
| Inflorescence | 77 | - | - | - | - |
| Inflorescence | 78 | - | - | - | - |
| Inflorescence | 79 | - | - | - | - |
| Inflorescence | 80 | - | - | - | - |
| Inflorescence | 81 | - | - | - | - |
| Inflorescence | 82 | - | - | - | - |
| Inflorescence | 83 | - | - | - | - |
| Inflorescence | 84 | - | - | - | - |
| Inflorescence | 85 | - | - | - | - |
| Inflorescence | 86 | - | - | - | - |
| Inflorescence | 87 | - | - | - | - |
| Inflorescence | 88 | - | - | - | - |
| Inflorescence | 89 | - | - | - | - |
| Inflorescence | 90 | - | - | - | - |
| Inflorescence | 91 | - | - | - | - |
| Inflorescence | 92 | - | - | - | - |
| Bias (%) | 5.23 |  |  |  |  |
| Stand.Dev.(%)  uLoA (%)  lLoA (%) | 5.14  15.30  -4.83 |  |  |  |  |
|  |  |  |  |  |  |

Sample no, number of sample; Conc UHPLC, THC concentration obtained by UHPLC; Conc UHPSFC, THC concentration obtained by UHPSFC; True value, mean between Conc UHPLC and Conc UHPSFC values; Relative difference, relative difference between Conc UHPLC and Conc UHPSFC; Bias, average of relative difference percentages; Stand. Dev., standard deviation of relative difference percentages; LoA (+), positive limit of agreement; LoA (-). negative limit of agreement.

**Tab. S3.** LLOD and LLOQ calculated for the five analytes quantified during the simulated routine use (THC, THCA-A, CBD, CBDA and CBN) with UHPLC and UHPSFC methods.

| Analyte | LLOD_UHPLC_  (µg mL^-1^) | LLOD_UHPSFC_  (µg mL^-1^) | LLOQ_UHPLC_  (µg mL^-1^) | LLOQ_UHPSFC_  (µg mL^-1^) |
| --- | --- | --- | --- | --- |
| THC | 0.11 | 1.56 | 0.34 | 4.72 |
| THCA-A | 0.52 | 3.23 | 1.58 | 9.78 |
| CBD | 0.33 | 2.30 | 0.99 | 6.96 |
| CBDA | 0.73 | 3.73 | 2.20 | 11.43 |
| CBN | 0.21 | 1.50 | 0.63 | 4.55 |

LLOD, lower limit of detection; LLOQ, lower limit of quantification.

**Tab. S4.** Randomized peak purity data from 33% of the sample set for the analytes quantified (THC, THCA-A, CBDA and CBN). Peak purity testing has been performed by Empower PDA software and with the following settings: wavelength limit, 200 – 400 nm; noise interval time, from 0.25 to 0.75 min; active peak region, 95%; purity passes, 1.

| Sample no | Analyte | Purity (component)  UHPLC-UV | Purity (component)  UHPSFC-UV |
| --- | --- | --- | --- |
| 1 |  |  |  |
|  | THC | 97.2 | 100.0 |
|  | THCA-A | 97.6 | 100.0 |
|  | CBD | - | - |
|  | CBDA | 99.5 | 100.0 |
|  | CBN | - | - |
| 4 |  |  |  |
|  | THC | 97.2 | 100.0 |
|  | THCA-A | 97.6 | 100.0 |
|  | CBD | - | - |
|  | CBDA | - | - |
|  | CBN | - | - |
| 7 |  |  |  |
|  | THC | 97.2 | 100.0 |
|  | THCA-A | 97.6 | 100.0 |
|  | CBD | - | - |
|  | CBDA | 99.5 | 100.0 |
|  | CBN | - | - |
| 10 |  |  |  |
|  | THC | 97.2 | 100.0 |
|  | THCA-A | 97.6 | 100.0 |
|  | CBD | - | - |
|  | CBDA | 99.5 | 100.0 |
|  | CBN | - | - |
| 13 |  |  |  |
|  | THC | 97.2 | 100.0 |
|  | THCA-A | 97.6 | 100.0 |
|  | CBD | - | - |
|  | CBDA | - | - |
|  | CBN | 100.0 | 100.0 |
| 16 |  |  |  |
|  | THC | 97.2 | 100.0 |
|  | THCA-A | 97.6 | 100.0 |
|  | CBD | - | - |
|  | CBDA | 99.5 | 100.0 |
|  | CBN | 100.0 | 100.0 |
| 19 |  |  |  |
|  | THC | 97.2 | 100.0 |
|  | THCA-A | 97.6 | 100.0 |
|  | CBD | - | - |
|  | CBDA | 99.5 | 100.0 |
|  | CBN | - | - |
| 22 |  |  |  |
|  | THC | 97.2 | 100.0 |
|  | THCA-A | 97.6 | 100.0 |
|  | CBD | 100.0 | 100.0 |
|  | CBDA | 99.5 | 100.0 |
|  | CBN | 100.0 | 100.0 |
| 25 |  |  |  |
|  | THC | 97.2 | 100.0 |
|  | THCA-A | 97.6 | 100.0 |
|  | CBD | 100.0 | 100.0 |
|  | CBDA | 99.5 | 100.0 |
|  | CBN | 100.0 | 100.0 |
| 28 |  |  |  |
|  | THC | 97.2 | 100.0 |
|  | THCA-A | 97.6 | 100.0 |
|  | CBD | - | - |
|  | CBDA | 99.5 | 100.0 |
|  | CBN | 100.0 | 100.0 |
| 31 |  |  |  |
|  | THC | 97.2 | 100.0 |
|  | THCA-A | 97.6 | 100.0 |
|  | CBD | - | - |
|  | CBDA | 99.5 | 100.0 |
|  | CBN | - | - |
| 34 |  |  |  |
|  | THC | 97.2 | 100.0 |
|  | THCA-A | 97.6 | 100.0 |
|  | CBD | 100.0 | 100.0 |
|  | CBDA | 99.5 | 100.0 |
|  | CBN | 100.0 | 100.0 |
| 37 |  |  |  |
|  | THC | 97.2 | 100.0 |
|  | THCA-A | 97.6 | 100.0 |
|  | CBD | - | - |
|  | CBDA | - | - |
|  | CBN | 100.0 | 100.0 |
| 40 |  |  |  |
|  | THC | 97.2 | 100.0 |
|  | THCA-A | 97.6 | 100.0 |
|  | CBD | - | - |
|  | CBDA | - | - |
|  | CBN | - | - |
| 43 |  |  |  |
|  | THC | 97.2 | 100.0 |
|  | THCA-A | 97.6 | 100.0 |
|  | CBD | - | - |
|  | CBDA | 99.5 | 100.0 |
|  | CBN | - | - |
| 46 |  |  |  |
|  | THC | 97.2 | 100.0 |
|  | THCA-A | 97.6 | 100.0 |
|  | CBD | - | - |
|  | CBDA | - | - |
|  | CBN | - | - |
| 49 |  |  |  |
|  | THC | - | - |
|  | THCA-A | 97.6 | 100.0 |
|  | CBD | - | - |
|  | CBDA | - | - |
|  | CBN | - | - |
| 52 |  |  |  |
|  | THC | 97.2 | 100.0 |
|  | THCA-A | 97.6 | 100.0 |
|  | CBD | - | - |
|  | CBDA | - | - |
|  | CBN | - | - |
| 55 |  |  |  |
|  | THC | 97.2 | 100.0 |
|  | THCA-A | 97.6 | 100.0 |
|  | CBD | 100.0 | 100.0 |
|  | CBDA | 99.5 | 100.0 |
|  | CBN | - | - |
| 58 |  |  |  |
|  | THC | 97.2 | 100.0 |
|  | THCA-A | 97.6 | 100.0 |
|  | CBD | - | - |
|  | CBDA | - | - |
|  | CBN | - | - |
| 61 |  |  |  |
|  | THC | 97.2 | 100.0 |
|  | THCA-A | 97.6 | 100.0 |
|  | CBD | - | - |
|  | CBDA | - | - |
|  | CBN | - | - |
| 64 |  |  |  |
|  | THC | 97.2 | 100.0 |
|  | THCA-A | 97.6 | 100.0 |
|  | CBD | 100.0 | 100.0 |
|  | CBDA | 99.5 | 100.0 |
|  | CBN | 100.0 | 100.0 |
| 67 |  |  |  |
|  | THC | 97.2 | 100.0 |
|  | THCA-A | 97.6 | 100.0 |
|  | CBD | - | - |
|  | CBDA | - | - |
|  | CBN | - | - |
| 70 |  |  |  |
|  | THC | 97.2 | 100.0 |
|  | THCA-A | 97.6 | 100.0 |
|  | CBD | - | - |
|  | CBDA | - | - |
|  | CBN | - | - |
| 73 |  |  |  |
|  | THC | 97.2 | 100.0 |
|  | THCA-A | 97.6 | 100.0 |
|  | CBD | - | - |
|  | CBDA | - | - |
|  | CBN | 100.0 | 100.0 |
| 76 |  |  |  |
|  | THC | 97.2 | 100.0 |
|  | THCA-A | 97.6 | 100.0 |
|  | CBD | - | - |
|  | CBDA | - | - |
|  | CBN | - | - |
| 79 |  |  |  |
|  | THC | 97.2 | 100.0 |
|  | THCA-A | 97.6 | 100.0 |
|  | CBD | - | - |
|  | CBDA | - | - |
|  | CBN | - | - |
| 82 |  |  |  |
|  | THC | 97.2 | 100.0 |
|  | THCA-A | 97.6 | 100.0 |
|  | CBD | - | - |
|  | CBDA | - | - |
|  | CBN | - | - |
| 85 |  |  |  |
|  | THC | 97.2 | 100.0 |
|  | THCA-A | 97.6 | 100.0 |
|  | CBD | - | - |
|  | CBDA | - | - |
|  | CBN | - | - |
| 88 |  |  |  |
|  | THC | 97.2 | 100.0 |
|  | THCA-A | 97.6 | 100.0 |
|  | CBD | - | - |
|  | CBDA | - | - |
|  | CBN | - | - |
| 91 |  |  |  |
|  | THC | 97.2 | 100.0 |
|  | THCA-A | 97.6 | 100.0 |
|  | CBD | - | - |
|  | CBDA | - | - |
|  | CBN | - | - |

Sample no, number of sample.
